# Editorial: Understanding emotions using brain imaging and stimulation techniques

**DOI:** 10.3389/fnhum.2023.1130140

**Published:** 2023-01-31

**Authors:** Alan N. Simmons, Irina A. Strigo

**Affiliations:** ^1^Veterans Affairs San Diego Healthcare System, Veterans Health Administration, United States Department of Veterans Affairs, San Diego, CA, United States; ^2^University of California San Diego Health, University of California, San Diego, San Diego, CA, United States; ^3^San Francisco Veterans Affairs Health Care System, Veterans Health Administration, United States Department of Veterans Affairs, San Francisco, CA, United States; ^4^School of Medicine, University of California, San Francisco, San Francisco, CA, United States

**Keywords:** emotion, brain imaging, brain stimulation, social, self

Humans are social animals. To understand their emotional status, it is important to comprehend how they relate to and perceive those around them. Much of the brain imaging research on emotion, as seen through the lens of scientific discovery and using the tools at our disposal, concludes the search for understanding emotion at the individual level. To truly appreciate and understand the anxiety experienced by these social animals, biomarkers and interventions need to expand beyond this limited frame of reference.

This Research Topic brings together a collection of manuscripts that look at novel approaches and pathways for resolving the conundrum of the individuals' isolating and often-anxious condition through the lens of their relationships with others. In their paper entitled “*TEAMwork: Testing emotional attunement and mutuality during parent-adolescent fMRI*,” Kerr et al. examined one of the most fundamental bonds: that between a parent and child. By performing simultaneous scanning and evaluation of this dyad, the researchers found that ventral prefrontal activation during collaborative tasks was associated with positive parenting practices. This suggests, in part, that the emotional regulation or empathy of a dyad can lead to positive parenting in partnership.

Conversely, in “*Right temporoparietal junction plays a role in the modulation of emotional mimicry by group membership*,” Peng et al. looked at the boundary state of ingroup and outgroup differentials in the context of emotional mimicry. In this work, they showed that ingroup biasing could be partially ablated by transcranial stimulation to the right temporoparietal junction, but it was not critical to mimicry in a broader context. Interestingly, this region has been shown to be important for self-concepts in relation to others.

A similar region was deemed important in the study by Nan et al., “*Neural dynamics during emotional video engagement relate to anxiety*.” This work looked at inter-subject correlations to determine the co-occurring activation in individuals watching the same movie. The response during a segment in which the clip's protagonist felt relief was of particular interest. This was related to activation in the superior parietal cortex, specifically, but not exclusively, in the theta band. This seemed to suggest this region is crucial to the shared social experience of seeing an affiliated other gain safety and the subsequent subsiding of anxiety.

These studies highlight the importance of a large network in the connection between humans, with the prefrontal cortex conceptualizing the other and the parietal cortex providing contextual information about this relationship to determine the subsequent, emotional impact on the self. However, in certain conditions, such as severe PTSD, the sense of self and the concept of self in relation to others can appear irrevocably altered. Lai et al. look at two such individuals in “*Acute effects and the dreamy state evoked by deep brain electrical stimulation of the amygdala: associations of the amygdala in human dreaming, consciousness, emotions, and creativity*.” In this work, they presented case studies of deep brain stimulation of the amygdala in two subjects with severe refractory PTSD. This research suggests that stimulation of the amygdala can induce strong and valenced emotional states, including dream-like or euphoric states. It is important to note that this work highlighted that the state induced was not uniform but showed substantial inter-subject variation across location and voltage. Positively induced states are often manifested as dream-like conditions, making the individual feel more engaged and attuned to these available affiliative states.

This series of articles shows the importance of understanding humans as social beings. Novel research can provide insight into the neural architecture that is needed to facilitate and maximize this state of connectedness. In combination, this work suggests that positive affiliative states can be instigated by stimulating the basolateral amygdala, that connectedness to those closest to us can be facilitated by prefrontal involvement, and that the contextualization of our relationships to a broader social structure can be manifested in the parietal lobe. This research parallels that on understanding of the self, as it is only when we juxtapose the self that we perceive ourselves in relation to others. The role of the parietal lobe may involve the separation of self and other within a conceptual space (Cavanna and Trimble, 2006). The role of the limbic cortex may relate to the sense of the self as an interoceptive entity (Craig, 2014; Seth, 2021). This Research Topic highlights that there is no single location in the human social animal that is uniquely responsible for emotions. These insights may pave the way for a more complex and informative model of the human as it exists as a social animal.

## Author contributions

AS and IS contributed to conceptualization, writing, and review of the manuscript. All authors contributed to the article and approved the submitted version.
